# Interobserver agreement in perineal ultrasound measurement of the anovaginal distance: a methodological study

**DOI:** 10.1007/s00192-017-3392-6

**Published:** 2017-06-17

**Authors:** Sofia Pihl, Eva Uustal, Linda Hjertberg, Marie Blomberg

**Affiliations:** 1 Department of Obstetrics and Gynaecology, Linköping University, SE-581 85 Linköping, Sweden; 2 Department of Clinical and Experimental Medicine, Linköping University, Linköping, Sweden

**Keywords:** Perineal ultrasound examination, Anovaginal distance, Perineum, Interobserver agreement, Validation

## Abstract

**Introduction and hypothesis:**

Objective outcome measures of the extent of laceration at delivery are needed. In this study we evaluated and describe here a method for learning perineal ultrasound measurement of the anovaginal distance (AVD). The learning period needed for examiners proficient in vaginal ultrasound examination and the interobserver agreement after reaching proficiency in AVD measurement were determined. The hypothesis was that the method is feasible to learn and reproducible for use in further research.

**Methods:**

The method was taught by an examiner experienced in perineal ultrasonography. The distance between the mucosal margin of the internal anal sphincter was measured with a vaginal probe. The studied examiners measured the AVD until similar results (±5 mm) were achieved. The AVD in 40 women was then measured and documented by two examiners who were blinded to each other’s results. Interobserver agreement was calculated using the kappa score.

**Results:**

Examiners with previous experience in vaginal ultrasonography had learned the method after performing five sets of comeasurements. The AVD measurements after the learning period showed almost perfect agreement (*κ* = 0.87) between the examiners.

**Conclusions:**

The method for perineal ultrasound measurement of AVD was learned quickly with high interobserver agreement. The method is feasible to learn and reproducible for use in further research.

## Introduction

Perineal lacerations after delivery are common and need careful evaluation and treatment to avoid significant morbidity. Current methods for pelvic floor evaluation after delivery are, however, subjective and vary in quality, and lacerations are often misdiagnosed and undertreated [1]. Objective, accessible and reproducible clinical examination methods for identifying the extent of lacerations could increase patient safety and facilitate comparative research [2].

The perineal tissue between the anal mucosa and the vaginal wall at the middle level of the anal canal consists of the anal mucosa, internal anal sphincter, external anal sphincter and the fibrous tissue that is the insertion point for the external sphincter, transverse perineal, bulbocavernosus and puborectalis muscles, as well as the rectovaginal fascia. In previous research, daily practice and reconstructive surgery, this tissue is referred to as the perineal body [3–5]. In more recent studies the perineal body is described in more detail using histopathology and various imaging modalities [6, 7]. Ultrasound can be used for evaluating the anatomy of the female pelvic floor [8–11]. Both endoanal and endovaginal 3-D ultrasonography require specific skills and equipment, today only found at specialized centres. In contrast, equipment for vaginal/perineal ultrasonography is available in all labour wards in Sweden and obstetricians/gynaecologists use it in daily practice. The perineal approach has been shown to be accurate for imaging the anal sphincters and the perineal tissue [12].

With a vaginal probe directed backwards from the distal vagina toward the anus, the anovaginal distance (AVD) can be measured [13]. The examination is quick and painless for the woman. If the AVD is short, some or all the perineal components are missing or thin [3]. As the AVD is a new concept, the relationship between the AVD and the perineal body is unknown. Whether or not the AVD could serve as a proxy indicator for obstetric perineal damage depends on the reliability of the measurement method. It has to be both easy to learn and reproducible to be generally useful in delivery wards. This study was designed to evaluate the use of perineal ultrasonography for measuring the AVD, in terms of both ease of learning by the examiner and interobserver agreement.

## Materials and methods

The study population was recruited from among women attending the gynaecological outpatient clinic of a university hospital between May 2013 and June 2014. Exclusion criteria were inability to understand spoken or written information. Printed study information was posted in the waiting-room. Examination of the study subjects included vaginal ultrasonography and had to be performed when two examiners were on duty on the ward at the same time. There was no selection regarding patient characteristics since the study objective was merely to evaluate the measurement technique. All women who were given written and verbal information about the study gave consent. Three examiners were chosen based on their different levels of experience of vaginal ultrasonography, which were 5, 15 and 21 years. As do most Swedish gynaecologists, they used vaginal ultrasonography in daily clinical practice to measure internal genitalia distances.

First, a pilot study was conducted to establish if the method was feasible to learn. We stipulated that five sets of comeasurements would be sufficient to learn the method. Five consenting women were measured each three times by the two examiners, one with and one without previous experience of AVD measurement. The technique was openly discussed and adjusted according to the instructions for measurement presented below. When the examiners produced three consecutive similar AVD values (±5 mm), they were considered proficient (data not shown). This was achieved after performing five sets of three measurements, and the method was considered robust enough for study of interobserver agreement.

All examinations were done with the woman in the lithotomy position as is standard in Swedish gynaecological practice. The equipment was a Bk Medical Flex Focus 500 1202 ultrasound scanner with a type 8819 9-5 MHz vaginal probe. The study was approved by the Ethics Review Board of Linköping University Hospital.

**Fig. 1:**
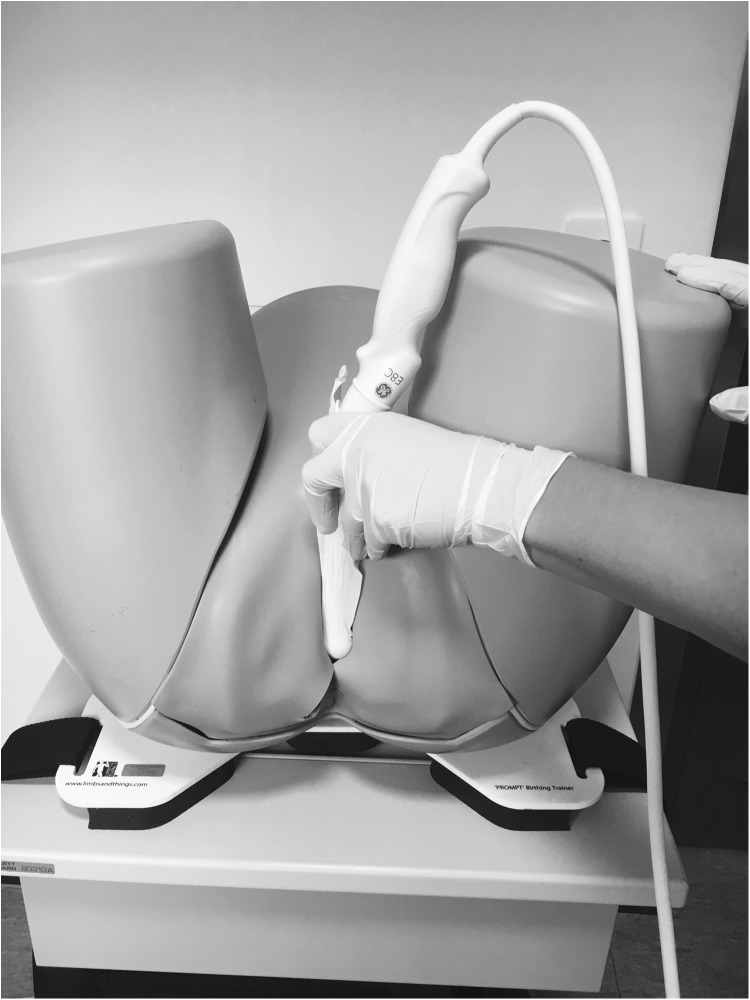
Technique for measuring the anovaginal distance

The measurement instructions were as follows:

1. Place the vaginal probe at right angles to the posterior distal vaginal wall in a transverse scanning plane (see Fig. 1).
2. Move the probe slowly cranially from the distal anal canal to the point where the internal anal sphincter first appears as a low-echogenic ring.
3. Adjust the image size so that the internal sphincter ring fills more than half the screen.
4. Steady the probe against the tissue using light pressure until the image begins to be distorted, then release the pressure until an undistorted image is just restored.
5. Freeze the image and measure the distance between the anal limit of the internal anal sphincter and the edge of the probe; this represents the AVD.
6. Repeat twice and record the measurements.

**Fig. 2:**
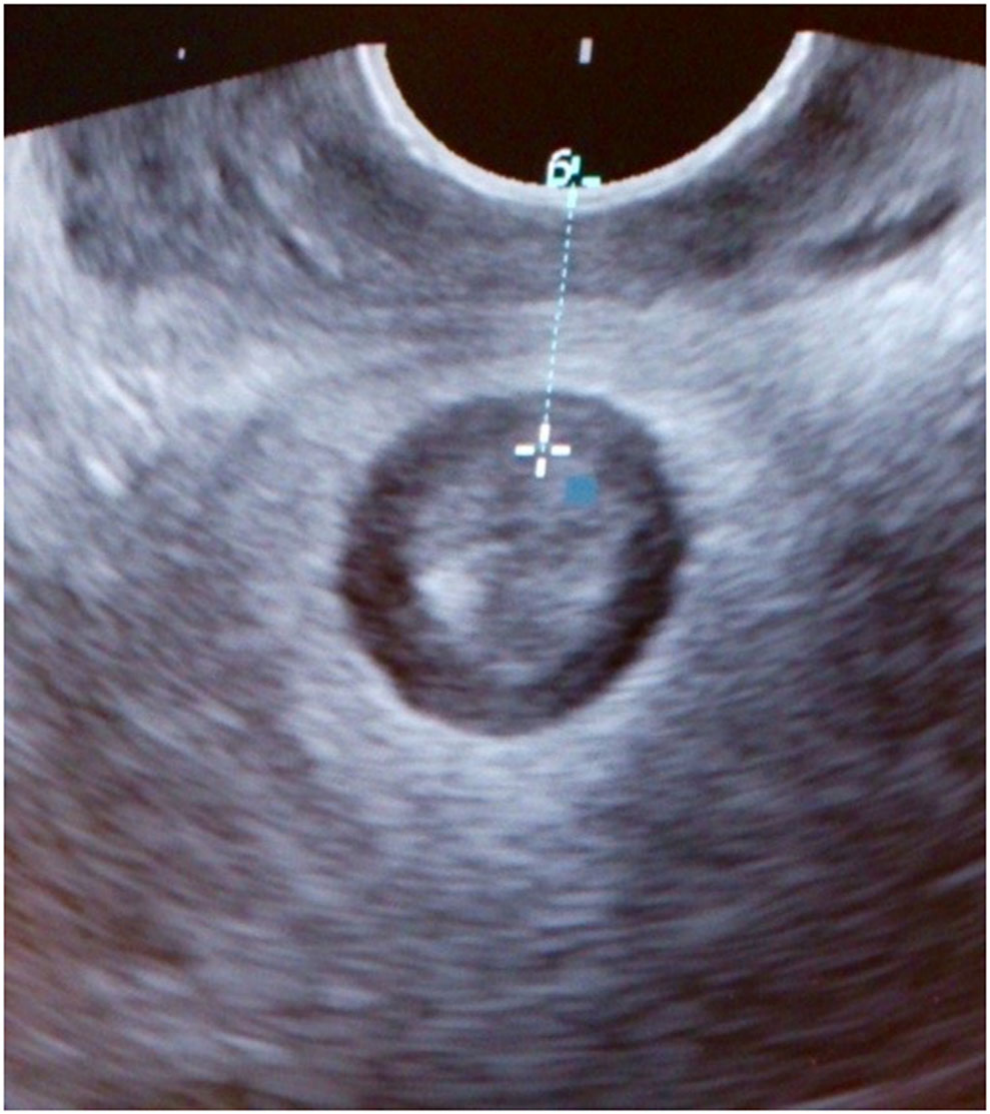
Perineal ultrasound image, obtained with a vaginal probe showing measurement of the anovaginal distance

Figure 2 shows the point of measurement defined as the mid-anal canal [14].

The first examiner made the first three measurements and silently documented them in the study protocol. The second examiner was then called in and made three measurements and documented them on a separate page of the protocol. The examiners did not see or hear each other’s results. In two women in whom the internal sphincter was torn and the limit could not be defined, the distance between the outer edge of the anal mucosa and the vaginal probe was measured. The mean AVD based on the three measurements obtained by each examiner were paired for every patient. Interobserver agreement was assessed using the weighted kappa coefficient and a Bland-Altman plot. Table 1 shows the definitions used to interpret the kappa coefficient [15, 16].

**Table 1:** Definitions of the levels of agreement in relation to the kappa coefficient (*ĸ*)

Kappa coefficient	Level of agreement
≤0.20	Slight
0.21–0.40	Fair
0.41–0.6	Moderate
0.61–0.80	Substantial
0.81–1.00	Almost perfect

Power calculations were based on data from a pilot study of the use of perineal ultrasonography for the measurement of AVD in five women performed by two examiners. The main study needed 40 women to have a power of 80% at a two-sided significance level of alpha = 0.05 with an accepted difference of 5 mm between examiners. The value of 5 mm was based on clinical experience. Forty women were recruited.

The statistical analysis was performed using Stata v. 13.1 (Statacorp LP, College Station, TX).

## Results

**Fig. 3:**
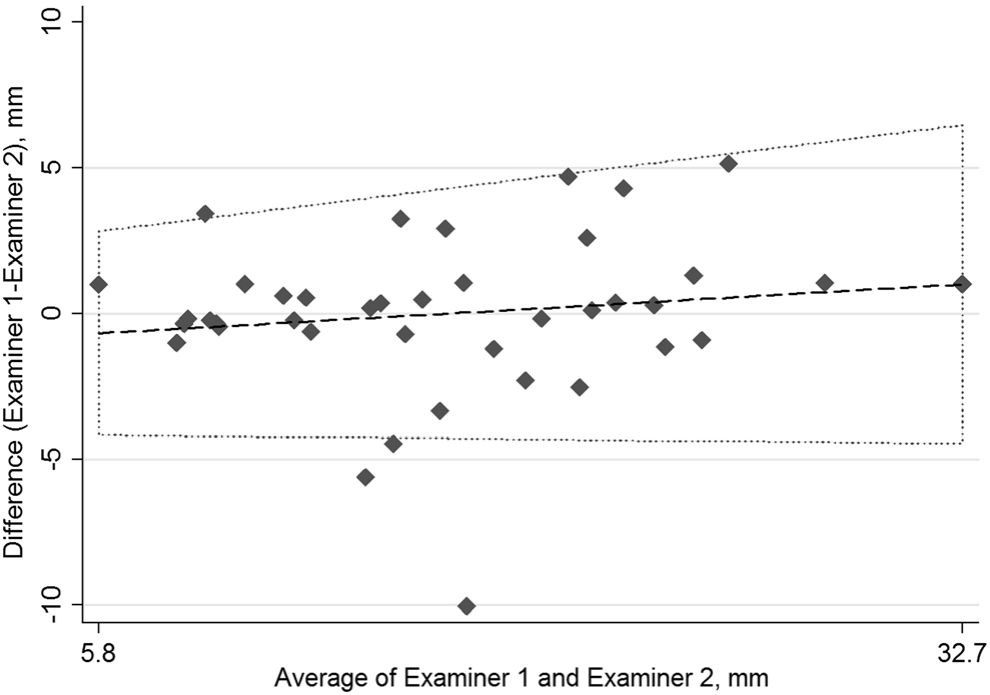
Bland-Altman plot of interobserver differences in measured anovaginal distance

A new examiner achieved proficiency after comeasuring five patients each three times. Three measurements were then made by both examiners in all 40 women. No discomfort was reported by the women. The mean difference in the AVD measurements was 1.8 mm (95% CI 1.13–2.45 mm, 99% CI 0.91–2.68 mm). There was one outlier of a difference of 10.1 mm, and three values more than the accepted difference of ≤5 mm between the examiners, all of which were included in the calculations. With an accepted difference of ≤5 mm interobserver variation, the weighted kappa coefficient was 0.87 (*p* ≤ 0.001) with an agreement of 92.5%, classified as almost perfect agreement (Table 1). Variation in the measurements was not significantly influenced by the length of the AVD. Interobserver variation is shown in Fig. 3.

The intraobserver variability ranged from 0.1 to 3.9 mm and there was no difference in variability in relation to years of experience. Examining systematic errors comparing mean measurements and standard deviations, there were no significant differences between individual examiners regardless of their experience with the use of vaginal ultrasonography in basic gynaecological clinical care (data not shown).

The mean age of the participants was 47 years (range 18–83 years) and their mean parity was 2 (range 0–5). Demographic data of the study population are presented in Table 2.

**Table 2:** Demographic data of the study population

Study protocol no.	Year of birth	Age (years)	Parity	Caesarean section	Anovaginal distance (mm)	Reason for visit	Additional medical information
1	1959	55	2	0	8	Lichen planus	Urinary incontinence, MP
2	1961	53	3	0	10	Rectocele	Faecal incontinence, TVT, MP
3	1959	55	2	0	21	Urinary urgency	TVT, MP, ERT
4	1931	83	2	0	9	Cystocele	MP, local ERT
7	1973	41	4	0	28	Cervical dysplasia	
8	1935	79	5	0	19	Cystocele	MP, local ERT
9	1961	53	2		23	Intrauterine device	MP
10	1988	26	0	0	17	Cervical dysplasia	
11	1975	39	2	0	25	Cervical dysplasia	
12	1974	40	1	0	20	Cervical dysplasia	
13	1952	62	1	0	18	Cervical dysplasia	MP
14	1938	76	2	0	16	Urinary incontinence	TAH + SOE, MP
15	1974	40	2	0	20	Urinary incontinence	
16	1979	35	3	0	33	Cervical dysplasia	
17	1967	47	2	0	25	Urinary incontinence	
18	1960	54	3	0	17	Cervical dysplasia	
19	1988	26	1	1	15	Urinary urgency	
20	1988	26	0	0	15	Cervical dysplasia	
21	1996	18	2	0	14	Cervical dysplasia	
22	1940	74	3	0	15	Urinary incontinence	Rectocele, cystocele, MP
23	1970	44	3	0	9	Bleeding	Myoma
24	1953	61	2	0	10	Pelvic pain	TAH, rectocele. MP
25	1972	42	3	1	12	Abdominal pain	
26	1957	57	0	0	12	Cervical dysplasia	MP
27	1980	34	2	0	12	Faecal incontinence	
28	1979	35	3	0	14	Pelvic pain	
29	1959	55	0	0	17	Urinary urgency	MP
30	1964	50	4	0	10	Rectocele	TVT, MP
31	1953	61	3	0	9	Rectocele	MP
32	1968	46	2	0	9	Cystocele	Rectocele, TVT
33	1995	19	0	0	16	Labia minora surgery	
34	1985	29	0	0	12	Miscarriage	
35	1989	25	1	0	6	Rectocele	
36	1975	39	3	0	22	Cervical dysplasia	
37	1963	51	2	1	21	Abdominal pain	
38	1992	22	0	0	15	Cervical dysplasia	
39	1965	49	3	1	22	Dysmenorrhea	
40	1953	61	3	0	21	Bleeding	
41	1951	63	2	0	24	Urinary incontinence	Rectocele, cystocele
42	1970	44	2	0	23	Intrauterine device	

*TVT* tension-free vaginal tape, *MP* Menopausal, *ERT* Estrogen replacement therapy, *TAH* Total abdominal hysterectomy, *SOE* Salpingo-oophorectomy

## Discussion

Perineal ultrasonography has a short learning period among examiners routinely performing vaginal ultrasound examinations. The method of measuring AVD evaluated in the present study seems to work and can be recommended for implementation in clinical practice. This is the first study of a method for teaching AVD measurement and the interobserver agreement among doctors with proficiency in vaginal ultrasonography. Interobserver agreement with the use of endovaginal 3-D ultrasonography of the pelvic floor has been shown to be good [17]. In studies of endoanal ultrasonography, a distance of less than 10 mm between the anal mucosa and the vaginal wall measured at the mid-anal canal level has been shown to be correlated with anal sphincter injury and anal incontinence [3, 18, 19]. Transperineal ultrasonography performed with a vaginal probe has been used to detect occult sphincter injuries directly after delivery [9]. Measurements of the perineal tissue components using this approach in the immediate postpartum period have not yet been reported.

The strengths of this study include the following, First, all measurements were made live in the clinical examination situation and not on images or video recordings analysed retrospectively, which indicates that the interobserver validity found is transferable to other clinical settings. Second, the women examined were not selected from a population with pelvic floor dysfunction, so that the examiners would have had no expectations of any ultrasound findings in the perineal area.

The study also had some limitations. The subjects were examined as gynaecological outpatients and not directly after delivery. For establishing reproducibility, it was considered more ethically sound to test the method in a calm setting. Also, all examiners were motivated to make the method work. How the teaching protocol will work among general staff in the delivery ward is now the subject of further studies.

There is an urgent need to establish objective outcome measures regarding perineal lacerations to evaluate preventative interventions and risk factors [2]. Even though postpartum endoanal ultrasonography of the perineal area shows missed lacerations and prevents anal incontinence [1], it is rarely used. The use of perineal ultrasonography for the measurement of AVD is less hindered by a lack of equipment and skills than endoanal ultrasonography. Having established its feasibility and reproducibility, we are now going on to evaluate perineal ultrasonography in clinical studies for the examination of women after delivery.

### Conclusions

Perineal ultrasound measurement of the AVD showed a short learning period for examiners with previous experience in ultrasound examination as well as a high interobserver agreement. The method described can be taught and reliably used in further research.
